# The impact mechanism of telework on job performance: a cross-level moderation model of digital leadership

**DOI:** 10.1038/s41598-024-63518-6

**Published:** 2024-05-31

**Authors:** Meihui Liao, Shiyuan Li, Hongda Liu

**Affiliations:** School of Economics and Management, Tongji University, Shanghai, 200092 China

**Keywords:** Telework, Job performance, Cross-level moderation model, Digital leadership, Job embeddedness, Job demands-resources theory, Health care, Health care economics

## Abstract

Traditional enterprise management believes that telecommuting activities are out of the enterprise's control, which may reduce staff performance. We use the extension of job demand-resource theory and work embeddedness theory to develop and test the intermediary mechanism of embedded in and out of work in telework. Moreover, it judges the mediating effect of job embeddedness on telecommuting → job performance. With the help of family conflict theory, we have revealed the possible performance changes in telework and the impact of family on telework. We predict embedding outside of work may reduce job performance. However, this worry will not happen under the adjustment of digital leadership and job insecurity. We collected survey data from 36 enterprise teams and 328 members. We have confirmed that work performance will not be reduced by telecommuting. Digital leadership magnifies the embedding of telecommuting resources into employees’ work to a certain extent and inhibits the embedding problem outside work caused by telecommuting requirements. The telecommuting requirement may become a positive factor for employees staying home and avoiding workplace conflicts. We confirmed the inhibitory effect of job embeddedness on turnover rate and expanded the antecedent model of job embeddedness theory.

## Introduction

The sudden outbreak of COVID-19 has changed the previous working norm: According to the national epidemic prevention policy, affected areas with confirmed COVID-19 cases often order standstill orders to reduce the movement of people. To avoid cross-infection, residents in affected areas must quarantine at home or in the isolation ward. These policy practices often result in companies being in a state of shutdown or abnormal operations, with employees only able to complete their job tasks in unconventional office spaces such as home or isolation wards. Because of the epidemic's complexity, expansion, and unpredictability, many firms have taken the initiative to propose a migration strategy for work scenarios to avoid the potential negative impact of disconnected operational links or temporary quarantine of essential employees. Companies are using telecommuting as the new normal to adapt to the pandemic's dynamic situation and maintain the continuity and stability of business operations.

In fact, from the employee's perspective, many employees believe that the freedom of telecommuting empowers them to do their jobs better^1^. In a familiar (home) or relatively quiet (isolated) space, employees avoid the undesirable distractions of colleagues and the strict control of leaders, thus enhancing their performance with a creative and motivated mental state^2^. However, previous research may suggest that there are some disadvantages to telecommuting^3^, such as prolonged telecommuting away from the work team, increased professional isolation that inhibits organizational identity, and, ultimately, employees who are chronically absent from the company may lose the motivation to improve performance. This difference in work form and outcome is a heterogeneous expression of job demands and resources. Job demands-resources (JD-R) theory suggests that the characteristics of any work form can be classified into two attributes: job demands and job resources^4^. Job demands are employees' physical, psychological, and social needs at work. In contrast, job resources are the resource provision activities that give employees positive incentives and guide them to reduce their workload and work positively, such as assistance in personal development and growth planning. The increase in work demands leads to tension or attrition in the work process for individuals, creating adverse effects such as job burnout and role conflict. In comparison, job resources are used as motivational elements to give employees a positive effect.

Therefore, according to JD-R theory, increasing telecommuting demands may lengthen employees' working hours and even intensify the conflict between family and work roles. At the same time, telecommuting employees with children at home may also need to deal with additional educational stress. This will undoubtedly cause a decrease in telecommuting efficiency^5^. However, at the level of work resources, the most significant impact of the epidemic is the exacerbation of employee loneliness under social and emotional isolation. The inhibition of work resources and the loss of motivation also amplify low job performance. However, the findings from Mockaitis AI suggest that this dissatisfaction and burnout may be eliminated if the digital divide is bridged and digital activities are introduced^6^. A possible situation is that, with the strengthening of modern digital capabilities, digital leadership replaces traditional leadership, allowing leaders to regulate telecommuting demands and resources with the help of digital activities^7^. For example, leaders use technologies for remote management, possibly through digital management technologies such as Ding Talk, Tencent meetings, and online punch-in to realize the supervision of employees’ work. Thus enhancing the current work demands or putting forward new demands. They may also realize online group building through digital leadership with the help of digital platforms, maintaining employees’ perception of a team atmosphere and eliminating the loneliness of being far from social and crowd, or enhancing information sharing and promoting knowledge circulation within the organization through digital leadership, thus amplifying the incentive of work resources. Thus, digital leadership moderates the characteristic effects of telework demands and resources.

Becker et al.^8^ has also addressed the uncertainty of telework and job performance as a dominant outcome of employee moods and perceptions. William J Becker explains that the impact of telework varies from person to person, with home working often having a negative impact when employees do not like to mix work and life. However, when employees clarify the boundaries between work and home, they may gain more autonomy in telecommuting. This reflects the employee’s connection to all work-related scenarios inside and outside the organization. This mediated activity of the employee’s mood and perception objectively represents work embeddedness. When employees are actively embedded in work activities and delineate work-life boundaries, or when employees feel the comfort and strong links of the work organization, they are in a state of on-the-job embedding and perceive more losses from leaving, thus focusing on work. On the contrary, employees may be forced into off-the-job embeddedness if there is an increase in life elements and extra-work connections. Based on the previous analysis, the increase in telework resources may allow employees to focus on activities on their jobs. Although on-the-job embeddedness may generate some isolation, a motivational framework can construct comfortable organizational links for employees, and work-focused activities are more likely to enhance work performance. On the other hand, the increased demands for telework may exacerbate employees’ work hours and amplify work-family conflicts in identifying family roles and arranging their lives. The increased connection with non-work factors amplifies off-the-job embeddedness and indirectly affects job performance. It is of concern that regardless of how family-work conflict reacts to the outcome of off-the-job embeddedness, no employee wants to lose his job during an epidemic. Thus, job insecurity may overcome work-family anxiety, forcing employees to focus on job performance as their only goal.

This study intends to explore the correlation mechanism between telework characteristics and job performance during COVID-19 to eliminate the wrong perception of telework as' Tang Ping' and' Mo Yu' in Chinese (which can be translated into ‘lying flat’ and ‘slaking off’ in English) at the social level and to guide leaders in reshaping their perception of telework management mode.

This paper dissects the mechanisms linking telecommuting to job performance during the epidemic, examining the mediating role of job embeddedness and the moderating role of digital leadership and job insecurity, with the following potential theoretical contribution and practical insights.

1. Firstly, discussing the telework phenomenon in COVID-19 enriches the application of job demands-resources theory to telework and brings this theory into the realistic context of COVID-19. It can solve more problems by applying enterprise theory in the COVID-19 crisis. Secondly, COVID-19 broadens the practical scenarios of work embeddedness theory, enriching the application possibilities of work embeddedness theory and interacting it with job demands-resources theory at multiple levels, extending the natural connection between the two theories. Finally, a moderating mechanism of digital leadership and work insecurity is established, which provides more theoretical thinking and inspiration for the subsequent normalization of epidemic prevention and control and normalization of enterprise operation.
2. *Practical Insights* This paper focuses on reversing the misconceptions of telecommuting at the societal level to serve the development strategies of companies and employees under the frequent existence of future epidemics. Employees can use digital leadership and job insecurity to regulate across layers, as well as the mediating research for the balance between on-and-off job embeddedness, to improve their productivity during the epidemic to adapt to the complex situation of future epidemic offices. For enterprises, this paper provides directions for improving multi-dimensional management approaches, such as telecommuting management and digital leadership design, to help enterprises develop as well as possible during the epidemic.

In addition, another possible contribution to this paper is that we hope that regardless of the epidemic, diversification of work styles is a vital tool to help employees and companies improve their performance and that employees can get more home and work output from telecommuting without being bound to a specific work scenario. We hope those office activities in China can be more liberalized so that we can agree to form the concept of ‘what suits you is the best and eliminate the traditional bad ideas of ‘what you cannot see is bad’ and ‘if you do things at home, you cannot do anything well.

## Literature review

### Demands and resources for telework: insights from job demands-resources theory

Whatever your nationality, work always seems boring^9^. Given more choices, people always prefer the latter between work and well-being. Work is seen as a deal tool: employees trade their time, energy, and emotions for salary, value, and self-efficacy. However, a boring job or a repeated arrangement in the long term always leaves employees with a distinct sense of burnout. Burnout reflects unreasonable work demands, such as overtime, compulsive socialization, etc.^10^, which will easily make employees feel that the "work deal" is unfair, namely, giving too much and getting too little. Bakker and Demerouti^11^, on the other hand, suggest that eliminating burnout requires investing work resources, i.e., work gives employees more salary or self-efficacy, such as reducing the psychological and physical costs that employees have to pay to work, which can be seen as care resources for employees^12^. Alternatively, work can give employees resources, such as encouraging them to train, providing support from colleagues and superiors, and helping them reach their work goals quickly. The changes in burnout come from the changing nature and balance of work transactions. When the demands of work increase on employees, they become more powerless in problems such as work overload and work-family conflicts^13^. On the other hand, job resources, such as compensation, positively affect employees. In essence, this evolved job demands-resources theory clarifies the dual path of 'gain' and 'loss' of job characteristics on employees' physical, psychological, and professional development.

During COVID-19, the work demand-resource conflict appears to be more pronounced. Wei reported that when employees were forced to take time off or telework, the individual energy consumed by work demands did not diminish^14^. Taking work home amplifies family anxiety and conflict for employees. With the intensification of the epidemic and the implementation of China's epidemic policies, telework, mainly from home, is the dominant mode of work. The emergent demands of telework have fueled the work-family conflict. On the one hand, married employees get more work stress at home than single people because they need to balance their spouse's life^15^. In addition, if there are older people or children in the family, they are required to take on more caregiving responsibilities. Although the company may not have changed the work hours, they are inadvertently taking on overtime demands due to family chores, and their working hours are becoming more fragmented^16^. In addition, employees may need to expend more energy to handle their work because of closer family communication. Moreover, these struggles are often not needed for them to handle during non-epidemic times. Educational institutions tutor children and the elderly and even prepare dinner for them^17^. On the other hand, the demands for telework increase the effort of employees due to the constraints of the workplace. Such as inconvenient access to information and difficulty in approving work often leaves employees in a negative mood of work overload^18^.

In addition, although we observe that work resources have compensatory and motivational effects, telework resources are narrowed compared to traditional work. For example, traditional work can give employees face-to-face care, a team atmosphere, and other resources^19^. However, it is undeniable that telework resources continue to affect employees, especially single ones positively. On the one hand, telework resources help employees to focus better on their work and promote personal learning. Even though these benefits are not observable in reality, their sense of comfort boosts performance^20^. On the other hand, telework resources promote the achievement of work goals through the close connection between work organization, work elements, and employees. Besides, employees' anxiety will be alleviated, and the harmful effects of work demands will be invariably addressed21. When leaders are willing to understand and care about employees' lives, their work-family conflicts will also be moderated. However, Gabriel AS argued that it is debatable whether the resource feature of telework can be successfully established^21^, as JD-R theory emphasizes that loneliness is a crucial motivator for employees' self-efficacy. The mechanism of work resources is to use the positive effects of resources to detoxify employees' loneliness and embed them in the organization and work (in fact, these views are also considered by some Chinese scholars as 'black-hearted' businessmen: the ultimate goal of leaders caring for their employees is to brainwash them to be more focused on their work). However, it has yet to be determined whether these telework resources will still play a positive role during the epidemic and whether the isolation of employees will be eliminated.

We draw new inspiration from Hobfoll^22^, who argued that the demands and resources of telework needed to be leveraged externally. Jia et al.^23^ argued that telework resources could still provide employee care if companies use digital tools to eliminate the feeling of distance caused by telework. Due to the hardships during the pandemic, the positive effects of telework resources will be amplified, and employee loyalty will be enhanced. We also found in the findings of Lal et al.^24^ that employees cannot even receive task assignments without digital tools for telecommuting needs. Therefore, the moderating role of digitalization, i.e., digital leadership, will be explained in detail later.

### Demonstration of digital leadership: a common characteristic of management and art

Leadership is a term at a broad level, considered an attributive outcome for followers^25^. Leadership influences employees when they identify with the leader’s behavior and follow the leader. It is also considered a formalized structure of power given to the leader by the company or organization. The process of managing, planning, and controlling the multi-functional activities of the leader over the employees is the process of leadership penetration^26^. These two levels of definition determine the differentiation between leaders and followers and indicate that the leader’s behavior can be observed, and this visible result is leadership^27^. In the twenty-first century, the emergence of advanced information technologies, such as the Internet, e-mail, video conferencing, and virtual teams, has enriched the results of leadership display.

On the one hand, how leaders manage their employees have diversified, and these technologies have mainly enhanced leaders’ control, as reflected in their use of technology. On the other hand, Avolio^28^, guided by adaptive structure theory, found that advanced technologies have also changed the stereotypical management perception of leaders. Since many information technologies cannot be separated from life, the use of these technologies also makes leaders unconsciously integrate into the lives of their employees. For example, if an employee posts a dynamic in the WeChat circle of friends and forgets to block the leader, the leader may also use this technology to ‘observe’ the life of employees. Conversely, employees can also ‘peek’ into the other side of the leadership in these technology platforms. Balan and Cavendish^29^ define this metamorphosis of leadership as e-leadership. At the managerial level, leadership began to be delivered through electronic technology; at the artistic level, leadership opened up scenarios outside work, such as WeChat groups, Zoom meetings, etc. The former is defined as the electronic transformation of leadership in the delivery mechanism, while the latter is seen as the addition of electronic communities to the sources of leadership^30^.

George C. Banks evolved the concept of digital leadership based on e-leadership^31^. The significant difference between the two is the blurring of the boundary between leaders and employees. This blurring does not mean that employees can reject the leader’s leadership or that the job content of the employee and leader is confused. Instead, digital leadership proposes it virtualized scenarios distinct from the natural environment^32^, which can bring employees and leaders closer together. This sense of distance can be characterized on both psychological and spatial levels. At the psychological level, Svescu et al.^33^ recognized that employees evaluate the signals the leader sends in the digital age. Employees may have many different needs that need to be attended to by their leaders. For example, leaders will be concerned about employees’ negative moods through the dynamics they post in their friend circle, which may lead them to offer care or help. In e-leadership, leaders are simply ‘watching’ and not acting. Thus, digital leadership perceives more of the needs of employees and optimizes the leadership and members exchange. While this ability still comes primarily from electronic technology, there is one more feedback path than the single-direction relationship of e-leadership, which is to listen to the needs of employees and give feedback with the help of digital technology. Thus, the delivery mechanism of digital leadership is multi-directional, and employees can objectively evaluate, observe, and feel it. Thus, the delivery mechanism of digital leadership is multi-directional, and employees can objectively evaluate, observe, and feel it. At the spatial level, digital leadership solves the problem of geographically separated, temporally asynchronous work and is fully mediated and optimized by digital technology. However, Matthews et al.^34^ argued that digital connectivity via Wechat or Zoom only partially solves the spatial problems associated with telework. They emphasized that the most significant contribution of digital leadership to eliminating spatial distance was the construction of virtual relationships between leaders-employees and virtualized work communities. In such communities, the rules of the traditional office space are maintained, but they are not limited by spatial geography. In other words, digital leadership builds a new virtual online office and maintains the influence and scope of traditional leadership. Thus, digital leadership is a rich source that demonstrates the art of leadership in a virtualized context. E-leadership, on the other hand, although it also builds electronic communities, is in reality presented only as workgroups or office groups^35^, in which a strong connection between leaders and employees is missing (in China, employees often prefer to open more groups behind the leadership’s back to facilitate their accessible communication without being scolded by the leader). However, leaders can often turn it into a warm community in digital leadership, for example, by putting their heads down and joking around or sending out red packets. Moreover, the virtualized communities built by digital leadership are not simply groups but more like corporate forums and larger community camps^36^.

Overall, digital leadership's managerial and artistic characteristics make it observable to employees. Digital leadership's optimization of the workspace also reinforces the possibility of telework during epidemic times. Since digital leadership has a two-way transmission channel to employees’ life and work demands, this makes us highly concerned about the mechanism of digital leadership’s influence on telework demands and resources. We will precisely dissect this potential relationship later.

### Job embeddedness: an auxiliary explanation of work-family conflict theory and the moderation of job insecurity

Mitchell first proposed job embeddedness to explain employee-initiated turnover^37^. Job embeddedness includes two dimensions: on-the-job and off-the-job^38^. On-the-job embeddedness indicates the extent to which employees are embedded with the organization, while off-the-job embeddedness indicates the embeddedness of employees’ links to family or community. Mitchell and Lee^39^ argued that employees avoid leaving voluntarily when attached to an organizational relationship. Employees tend not to leave voluntarily when they cannot accept the loss^40^. Sekiguchi et al.^41^ further found that when employees do not want to leave their jobs due to a high level of on-the-job embeddedness, they need to work hard to improve their performance to gain leaders’ recognition. Karatepe and Osman^42^ argued that employees with a high level of on-the-job embeddedness usually would not leave their jobs voluntarily, while the effect of off-the-job embeddedness is multifaceted. For example, employees who are poor and have to take more responsibility for caring for families tend to show higher levels of off-the-job embeddedness. However, they are less likely than others to leave voluntarily because of their family responsibilities^43^.

Moreover, for employees who are more concerned about themselves, it is possible that the excessive embedding of the job has instead boosted the willingness to leave voluntarily. In the context of the recession, it is evident that most employees do not want to be dismissed from their companies. Although this appears to be the result of the influence of environmental variables, based on job embeddedness theory, it can be deduced that employees are reluctant to leave their jobs due to the deteriorating economic environment and the simultaneous increase in on-and-off job embeddedness. As a result, employees also strive to improve their job performance^44^. However, this path is undoubtedly moderated by employee insecurity and shows various possible evolutionary trends. On the one hand, as employees become more embedded in their jobs, their expectation of improving their performance to gain firm recognition increases. However, suppose employees are confident in their performance and do not have a sense of crisis that they will be dismissed. In that case, low levels of insecurity do not stimulate the mechanism of embeddedness^45^. For example, some employees with privileged backgrounds do not care about their jobs and do not have job insecurity, even if they have much pleasure at work or have developed close ties with colleagues. They tend to retain their usual work attitude, so job insecurity does not moderate their performance. On the contrary, if the employees depend on their jobs for livelihood, this insecurity may stimulate positive performance embedded in the job, which leads to higher job performance^46^. On the other hand, job insecurity may be the last straw that drives employees to leave if they love their families or are deeply tied to them. Jiang et al.^47^ concluded that job externalization is a passive employee stimulus that forces them not to leave their jobs. Furthermore, suppose job insecurity breaks through the threshold of their idea of leaving. In that case, it is possible to make them let go of the idea of leaving or ultimately reduce their job performance. Of course, the study by Brooks et al.^48^ also concluded that if job insecurity is more stable, it is possible to inhibit the negative impact of off-the-job embeddedness on job performance.

Some scholars have paid attention to other possible effects of off-the-job embeddedness. M. R. et al. introduced the work-family conflict theory in their study^49^. The study argues that off-the-job embeddedness not only manifests as an intensification of employees’ ties to their families or communities but is also essentially the result of employees’ family-work conflict afterward. Most employees shift from on-the-job to off-the-job embeddedness in epidemic and telecommuting environments. Inconsistent with the non-epidemic period, the mechanism by which previous off-the-job embeddedness reduced turnover was that increased family responsibilities discouraged employees from leaving voluntarily.

In contrast, under the epidemic cycle, the mechanism of action of off-the-job embeddedness is adjusted to become that due to telecommuting, employees are forced to have closer ties with their families than before, such as being more embedded in off-the-job scenarios (family activities, nucleic acid tests or epidemic prevention activities, etc.)^50^. Thus, the on-the-job embedded group may contain some employees who previously wanted to leave. Despite a lower voluntary turnover rate due to the COVID-19 outbreak, it may reduce job performance and negatively affect the company's development. Therefore, regulating the degree of off-the-job embeddedness in this state is essential.

## Hypothesis development

### Telework demands-resources and job embedness: moderation of digital leadership

Based on the analysis above, the job demands-resources theory is based on a balancing perspective to explain the enrichment process and conflict between job demands and resources. This theoretical model describes how job demands and resources affect individuals' feelings about their jobs and their families' outcomes. In Chinese companies, managers often complain about employees' positive attitudes toward work. Chronic employee burnout and inefficiency are difficult for companies to accept. Employees perceive a depletion of physiological and psychological future development effectiveness under work stress^51^. Grandey and Cropanzano^52^ argued that the imbalance in these firms resulted from the absence of resource preservation activities under the conflict between work demands and resources. Resource preservation theory emphasizes that the primary human motivation is to create, maintain, or gather resources and that resources are the most valuable things people have. Therefore, an increase in work demands essentially removes the regular resources belonging to employees and forces them to devote these physical or psychological resources to work. On the other hand, work resources divide the company's resources among employees to replenish their resource reserves and relieve their work fatigue.

Interestingly, Marco's study found that increasing work demands while forcing employees into work did not increase on-the-job embeddedness^53^. There are two possible reasons for this: the employees’ long-standing rebelliousness. Alternatively, job burnout due to job demands makes employees want to stay away from work more or not want to form a close work connection. The other possibility is that the increased work demands are breaking the peace in employees’ lives, and conflicts between family and community bother employees. For example, spouses often complain that their husbands spend too much time on work functions or business trips, which leads many husbands to try to compensate by taking time away from work for the family. Alternatively, these husbands separate their public and private affairs and maintain a limited and absolute association with the organization. The study by Demerouti et al.^54^ added that when the job requires the diversion of resources from the employee to the job, this is also a manifestation of the violation of the employee's life. Thus Gu and Wang^55^ argued that the increase in work demands could cause employees to amplify family-work conflicts and have to perform some family and community bonding maintenance work.

In contrast, Braunstein-Bercovitz found that increased work resources led employees to be more focused and love their work, maintaining high-level on-the-job embeddedness. This may be explained by the fact that work gives employees value recognition and cuts across their personal physical, psychological, and self-efficacy realizations. Employees are more willing to invest in their work. Of course, there is also the view that because companies treat their employees so well, families or communities should also support employees in giving back to firms.

In the previous analysis, we applied the work-requirement-resource theory well to the telecommuting problem. We found that the demand-resource properties of telecommuting have mostly stayed the same from the traditional theory. Therefore, there may also be a positive association between telecommuting resources for job embeddedness and telecommuting demands for off-the-job embeddedness. Moreover, Chong et al.^56^ added to the analysis of the epidemic the special consideration of telecommuting: since telecommuting directly integrates the work environment with life. This in itself is an intangible work demand for employees. This is because the reality is that more employees may want to get time off (especially in China) because of the epidemic rather than being isolated or telecommuting from home activities. In some companies, employees are not getting full pay because of telework, which they see as an apparent telecommuting demand. The need to telecommute amplifies the home-work conflict, forcing employees to distract themselves from their work.

Moreover, employees gradually seek to disengage from work embeddedness in this work stress and burnout^57^. Employees may try to close themselves off by turning off their cell phones or doing as little work as possible. This leads us to the hypothesis that there is a positive correlation between telework demands and job embeddedness.

In contrast, the telecommuting resources of the company during the epidemic activity broadly activate the employees' job embeddedness. A few research studies based on the psychology of employees during the epidemic show that employees tend to be more engaged in their work if the company is caring at all times during the epidemic rather than rushing work^58^. Moreover, elements such as understanding of employee interactions with other colleagues, greetings from leaders and even tilting of benefits, and corporate encouragement of employees to use isolation or home time to recharge for self-development also make employees more engaged in their work and take away the pain of emotional isolation in COVID-19. Resources for telecommuting may be positively correlated with job embeddedness. In summary, we propose the following hypothesis.

- *H1* Telework resources are positively correlated with on-the-job embeddedness
- *H2* Telework demands are positively correlated with off-the-job embeddedness

Unlike working during non-epidemic periods, both the resources and the telecommuting demands require digital leadership regulation. On the one hand, the supply of resources and the demand for work need to be released through leaders based on digital technology—for example, telecommuting resources in which concerns from superiors can often be delivered only through digital platforms. Scheduling some work requirements relies on platforms such as Ding Talk to unfold. Thus, the management activities of digital leadership are closely linked to telecommuting activities. These are the leaders' use and impact on digital technology.

On the other hand, the artistry of digital leadership moderates telecommuting activities. Magesa and Jonathan^59^ argue that this artistry manifests digital leadership after inheriting the charismatic attributes of leadership, i.e., the impact of digital technology on leadership, as analyzed in the previous article. In telecommuting resources, employees' burnout and complaints about their work are fed back to the company through digital technology, which gives more opportunities for leadership to be used. In telecommuting requirements, some work and family conflicts, employees often produce some work adjustments even after being noticed by the leaders. Based on digital leadership theory, we assume that digital leadership is a specific leadership resource that belongs to the organizational level. It explains the relationship between telecommuting activities, requirements, and resources that generate employee resource changes, and these changed resources serve their work or family. Digital leadership, as a cross-level resource, can complement the loss of employee resources due to telecommuting demands and amplify the stimulating effect of telecommuting resources on job performance^60^. Organizational and artistic activities of digital leadership make employees more active and coordinate their lives, so the results of this resource compensation or output change the employee job embeddedness. This leads us to hypothesize that:

- *H3* Digital leadership acts as a positive moderator between telework resources and on-the-job embeddedness
- *H4* Digital leadership acts as a negative moderator between telework demands and off-the-job embeddedness

### The relationship between job embeddedness and job performance: the moderation of job insecurity

Combined with the previous analysis, it is evident that job embeddedness often determines the likelihood of active separation^61^. However, in the context of the epidemic, often no employee wants to leave voluntarily, so job embeddedness is more likely to impact performance than cause willingness to leave^62^. When analyzed through traditional job embeddedness theory, embedding activities, both on and off the job, impact the willingness to leave voluntarily. It is usually the case that a higher level of embeddedness leads to a lower willingness to leave voluntarily. This logic applied to job performance research may lead to the conclusion that a higher degree of embedding on and off the job leads to better job performance^63^. However, during COVID-19, this changes: employees who are highly engaged in their work are more likely to perform better. On the one hand, they do not want to leave their jobs, and the high degree of embeddedness in their job often indicates that their lives are also work-centric. Therefore, they will focus on work performance and see it as a measure of personal development. On the other hand, combined with the results of telecommuting resources, the company's high level of commitment to such employees and the friendly relationships among colleagues contribute to their embeddedness with their work, and these positive work factors will make them give back to the company^64^. Of course, their reluctance to be asked by the firm during the epidemic will motivate them to perform better to avoid terminal elimination. Moreover, employees who are embedded outside work too much may have differential outcomes.

Guided by the family-work conflict theory, off-the-job embeddedness feeds back the value and connection they place on their families, and they are compelled to work hard for their families^65^. However, in the context of the epidemic, it is difficult for them to have the energy to work well after telecommuting switches the work scenario to home. On the one hand, they have to suffer from the contradictions of working at home. For example, they must coordinate with their spouses, elders, and children. Moreover, they must struggle for these family members (another manifestation of off-the-job embeddedness). These struggles add to the stress and exhaustion of employees. Even if they are reluctant to leave and would like to perform better, the objective result may be harsh: they cannot do their job well^66^. On the other hand, the attribution theory proposed by Kelley suggests that people usually make inferences and interpretations of employee behavior^67^. Therefore, the reasons for poorer employee performance in the context of the epidemic are attributed to family-related factors. Factors include emotions, family motivation, family roles, and personality^68^. In other words, we accept that we are all living worse and working harder than usual during the epidemic. Attribution theory tells us that we should attribute these factors to the family. After all, the result of on-the-job embeddedness should be to show better performance. However, when employees show worse job performance, we need to give a possible reason: off-the-job embeddedness. Of course, this paper does not deny that off-the-job embeddedness may also lead to other changes in performance. For example, you will work harder in an epidemic to provide for your family^69^. In reality, however, this paper, in conjunction with the social survey results, suggests that off-the-job embeddedness leads to worse employee performance. This is because frequent family conflicts make it difficult to do the job well. In summary, we propose that:

- *H5* On-the-job embeddednes has a positive relationship with employee job performance
- *H6* Off-the-job embeddedness has a negative relationship with employee job performance

Further, the following hypotheses were enriched by combining the telecommuting demands-resources and job embeddedness linkages from the previous analysis:

- *H7* There is a mediating role of on-the-job embeddedness between telework resources and job performance
- *H8* There is a mediating role of off-the-job embeddedness between telework demands and job performance

Furthermore, combining theoretical exploration and the real-world impact of the epidemic, we argue that job insecurity moderates the relationship between job embeddedness and job performance. Indeed, this job insecurity feeds into the fear of job termination during the epidemic^70^. Leaving a job during an epidemic can make it more difficult for people to survive. Thus, job insecurity inadvertently’ forces’ people to work hard^71^. This led to the following hypothesis:

- *H9* There is a positive moderating effect of job insecurity between on-the-job embeddedness and job performance
- *H10* There is a negative moderating effect of job insecurity between off-the-job embeddedness and job performance

**Figure 1:**
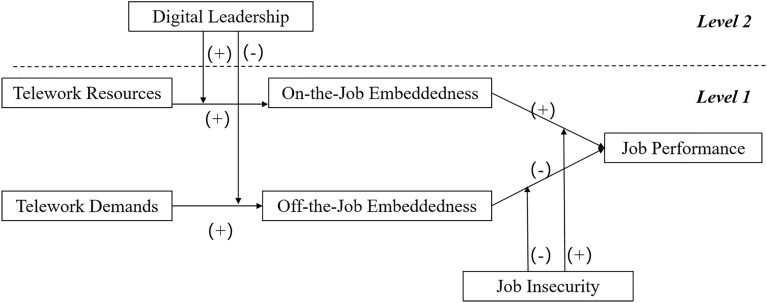
Theoretical framework.

Finally, we develop the following theoretical model (Fig. 1). We will test the hypothesis through various methods, such as questionnaires, and conclude a reasonable solution for companies and employees facing telework problems during COVID-19.

## Analytical approach

We tested the hypotheses by multilevel regression analysis in Mplus 8. Model 1 and Model 3 tested the main effect of the independent variable telework resources and demands on on-and-off the job embeddedness. Then, we added moderator digital leadership and its interaction term with independent variable to Model 2 and Model 4 (both are moderation hypotheses). Hypothesis 1–4 were tested on Model 1–4. In the same way, we tested the moderating effect of job insecurity on the relationship between on-and-off job embeddedness and job performance in Models 7, 9, 10, and 12 (Hypothesis 5, 6, 9, 10). The significance of change in squared multiple correlation was assessed at each step. We centered all independent variables used in interactions before forming interaction terms to reduce multicollinearity. To test Hypothesis 7 and 8 (mediation hypotheses), we also use Sobel tests and bootstrapping confidence intervals to test the indirect effect. The indirect effect is considered to be significant when the Sobel test t-value is significant (t > 1.96). When the bootstrapped confidence intervals (CIs) do not contain value 0, the indirect effect differs from 0. In our regression analysis, we controlled for age, gender, and education. Table 1 shows the means, standard deviations, correlations, and reliability.

**Table 1:** Descriptive statistics, correlations and reliabilities.

Variable	M	SD	1	2	3	4	5	6	7	8	9
Individual level											
1. Gender^a^	.49	.50									
2. Age	34.81	7.68	.25**								
3. Education^b^	2.15	.85	− .09	.15**							
4. TR	4.59	1.64	− .17**	.22**	.10	(.93)					
5. TD	5.34	1.37	− .02	.29**	.09	.18**	(.95)				
6. ONJE	4.85	1.42	− .21	.31**	.04	.22**	.25**	(.96)			
7. OFFJE	5.25	1.29	.00	.23**	.05	.08	.29**	.26**	(.95)		
8. JI	3.78	1.74	− .32**	.19**	− .03	.47**	.04	.28**	− .23	(.94)	
9. JP	5.04	1.45	− .24**	.33**	.04	.06	.35**	.43**	.34**	.11	(.91)
Team level											
1. Team size	9.63	3.12	.48**								
2. DL	5.27	1.38	.01	(.93)							

*N (employees)* = *308; N (groups)* = *32*.

*TR* telework resources, *TD* telework demands, *ONJE* on-the-job embeddedness, *OFFJE* off-the-job embeddedness, *JI* job insecurity, *JP* job performance (manager-rated), *DL* digital leadership. Standardized internal consistency reliability estimates (alphas) appear in parentheses along the diagonal.

^a^Dummy coded: 0 = female, 1 = male.

^b^Dummy coded: 1 = Specialty, 2 = Undergraduate, 3 = Master, 4 = Ph.D.

*p < .05; **p < .01; ***p < .001.

## Results

Table 2 displays the results of multilevel regression analyses. Hypothesis 1 and 2 proposed that telework resources and demands positively correlate with on-the-job and off-the-job embeddedness, respectively. While Model 1 and Model 3 show a significantly positive effect of telework resources on on-the-job embeddedness and telework demands on off-the-job embeddedness (*b* = 0.14, *SE* = 0.45, *p* < 0.05; *b* = 0.23, *SE* = 0.05, *p* < 0.001). Hypothesis 5 and 6 proposed that on-the-job and off-the-job embeddedness positively correlate with job performance, respectively. While Model 7 and Model 10 show a significantly positive effect of on-the-job embeddedness on job performance and off-the-job embeddedness on job performance (*b* = 0.35, *SE* = 0.05, *p* < 0.001; *b* = 0.32, *SE* = 0.06, *p* < 0.001). Thus hypothesis 1–2 and 5–6 were supported.

**Table 2:** Regression analysis for digital leadership and job insecurity as the moderator, on and off-the-job embeddedness as the mediator.

Variables	ONJE		OFFJE		JP					
	Model 1	Model 2	Model 3	Model 4	Model 7	Model 8	Model 9	Model10	Model11	Model12
Intercept	2.88*** (.45)	4.77*** (48.45)	2.97*** (.42)	5.21*** (.08)	2.29*** (.44)	2.45*** (.45)	3.70*** (.61)	2.26*** (.46)	1.67*** (.47)	3.56*** (.74)
Independent variables										
Telework resource (TR)	.14* (.05)	.11* (.05)				− .07 (.05)				
Telework demand (TD)			.23*** (.05)	.24*** (.06)					.26*** (.06)	
Mediator variables										
On-the-job (ONJE)					.35*** (.05)	.36*** (.05)	.07 (.12)			
Off-the-job (OFFJE)								.32*** (.06)	.26*** (.06)	.05 (.14)
Moderator variables										
Digital leadership(DL)		.30*** (.07)		.17** (.05)						
Job insecurity (JI)							− .48** (.15)			− .35* (.14)
Interaction										
TR × DL		.10*** (.02)								
TD × DL				− .09*** (.01)						
ONJE × JI							.12** (.04)			
OFFJE × JI										.11* (.04)

*N (employees)* = *308; N (groups)* = *32*.

*TR* telework resources, *TD* telework demands, *ONJE* on-the-job embeddedness, *OFFJE* off-the-job embeddedness, *JI* job insecurity, *JP* job performance (manager-rated), *DL* digital leadership.

Model reflects unstandardized regression coefficients with standard errors in parentheses.

*p < .05; **p < .01; ***p < .001.

**Figure 2:**
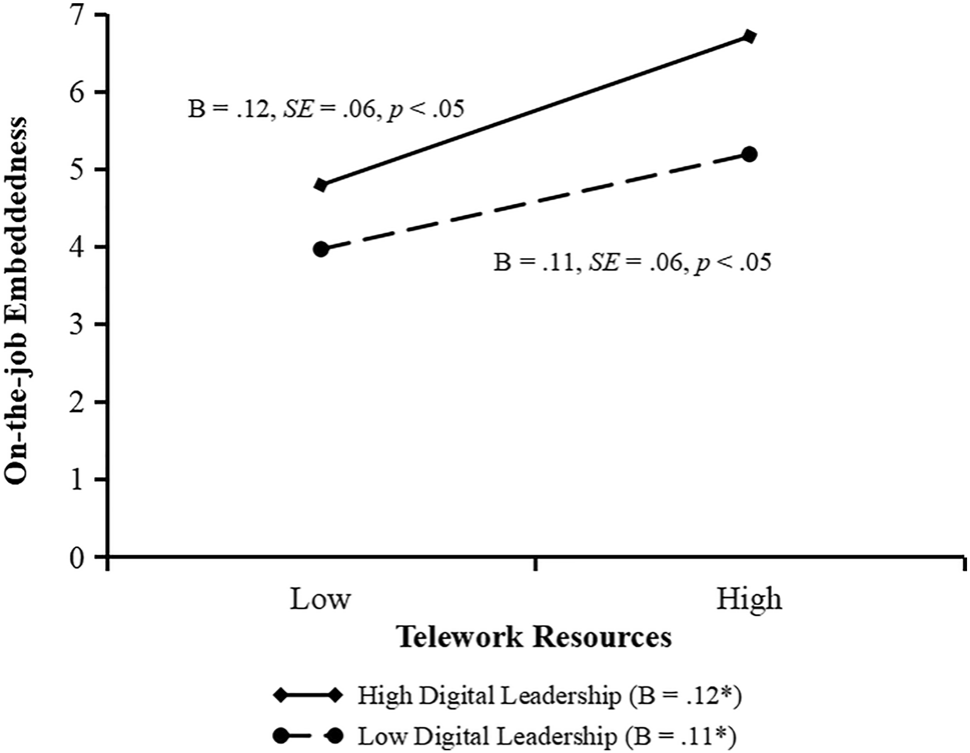
Moderating effect of digital leadership on the relationship between telework resources and on-the-job embeddedness.

**Figure 3:**
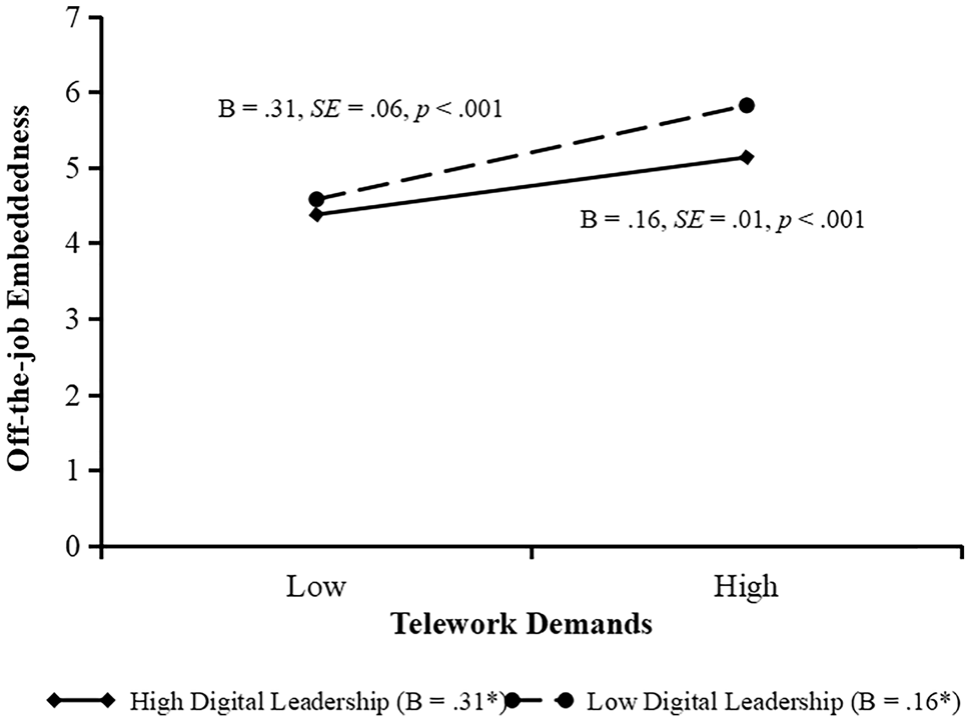
Moderating effect of digital leadership on the relationship between telework demands and off-the-job embeddedness.

As indicated in Model 2 and 4, the effect of the interaction of digital leadership and telework resources on on-the-job embeddedness and interaction of digital leadership and telework demands on off-the-job embeddedness is significant (*b* = 0.10, *SE* = 0.02, *p* < 0.001; *b* = − 0.09, *SE* = 0.01, *p* < 0.001). We plot these interactions by conducting simple slope tests. Figure 2 shows that the relationship between telework resources and on-the-job embeddedness is significant when digital leadership is is high (+ 1 standard deviation, simple slope = 0.12, *SE* = 0.06, *p* < 0.05), but the relationship becomes weaker when digital leadership is low (− 1 standard deviation, simple slope = 0.11, *SE* = 0.06,* p* < 0.05). Figure 3 shows that the relationship between telework demands and off-the-job embeddedness is significant when digital leadership is low (− 1 standard deviation, simple slope = 0.31, *SE* = 0.06, *p* < 0.001), but the relationship becomes weaker when digital leadership is high (+ 1 standard deviation, simple slope = 0.16, *SE* = 0.01,* p* < 0.001). The significant, positive interaction suggests that hypothesis 3 was supported, while the significant, negative interaction suggests that hypothesis 4 was supported.

**Figure 4:**
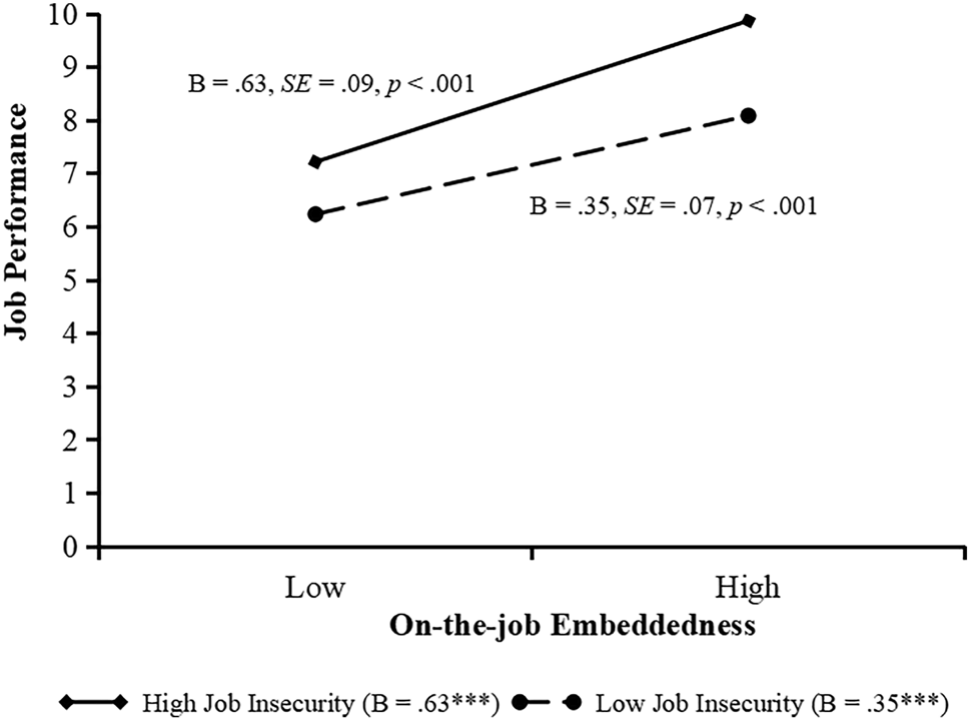
Moderating effect of job insecurity on the relationship between on-the-job embeddedness and job performance.

**Figure 5:**
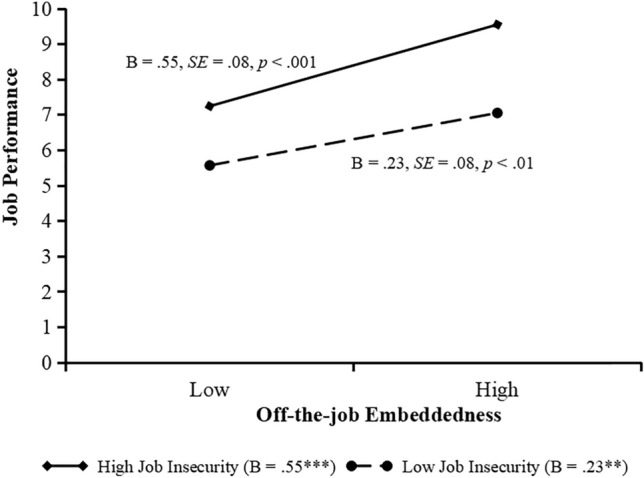
Moderating effect of job insecurity on the relationship between off-the-job embeddedness and job performance.

As indicated in Model 9 and 12, the effect of the interaction of on-the-job embeddedness and job insecurity on jobperformance and the interaction of off-the-job embeddedness and job insecurity on jobperformance is significant (*b* = 0.12, *SE* = 0.04, *p* < 0.01; *b* = 0.11, *SE* = 0.04, *p* < 0.05). We plot these interactions by conducting simple slope tests. Figure 4 shows that the relationship between on-the-job embeddedness and job performance is significant when job insecurity is high (+ 1 standard deviation, simple slope = 0.63, *SE* = 0.09, *p* < 0.001), but the relationship becomes weaker when job insecurity is low (− 1 standard deviation, simple slope = 0.35, *SE* = 0.07,* p* < 0.001). Figure 5 shows that the relationship between off-the-job embeddedness and job performance is significant when job insecurity is high (+ 1 standard deviation, simple slope = 0.55, *SE* = 0.08, *p* < 0.001), but the relationship becomes weaker when job insecurity is low (− 1 standard deviation, simple slope = 0.23, *SE* = 0.08,* p* < 0.01).

The significant, positive interaction suggests that hypothesis 9, 10 was supported.

To further test the mediation hypotheses, we performed the Sobel test.

**Figure 6:**
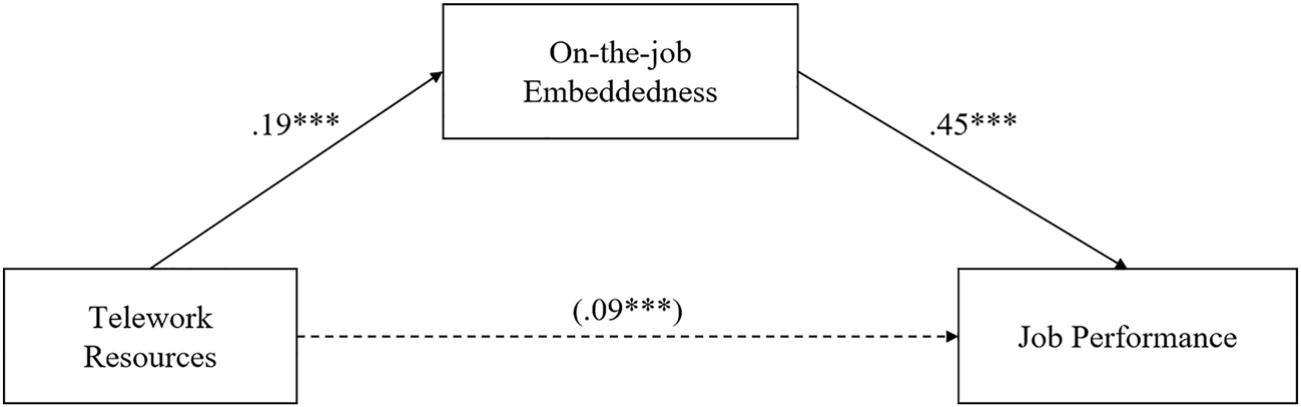
Mediating effect of on-the-job embeddedness on the relationship between telework resources and job performance. *Notes*:* n* = 308; In our regression analysis, we controlled for the age, gender and education. Type of mediation: full; Sobel t-value = 3.57^***^; Correlations in parentheses indicate indirect influence. *p < .05; **p < .01; ***p < .001.

**Figure 7:**
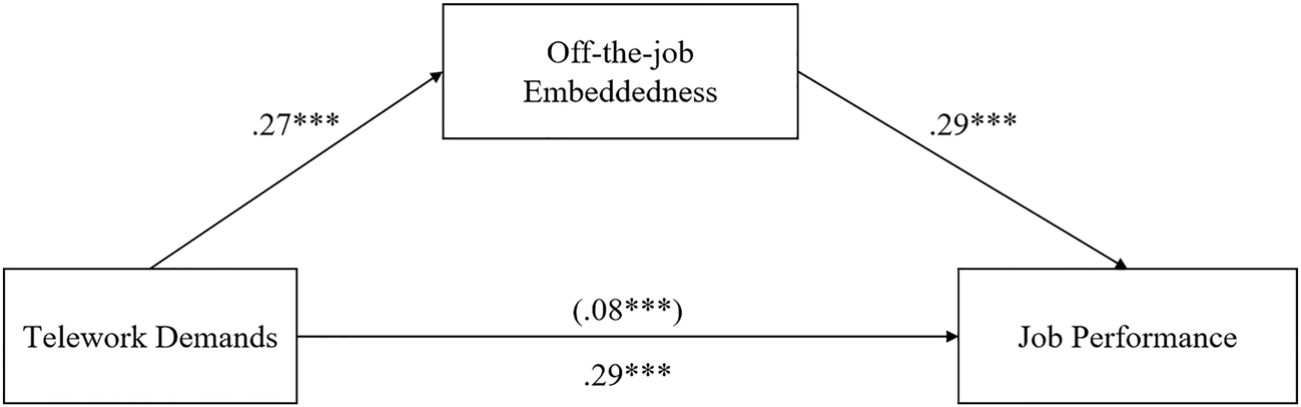
Mediating effect of off-the-job embeddedness on the relationship between telework resources and job performance. *Notes*:* n* = 308; In our regression analysis, we controlled for the age, gender and education. Type of mediation: partial; Sobel t-value = 3.50^***^; Correlations in parentheses indicate indirect influence. *p < .05; **p < .01; ***p < .001.

The result of Sobel test provided significant evidence of the existence of an indirect effect (t = 3.57, *p* < 0.001; 3.50, *p* < 0.001, Table 3). The bootstrap approach, making no assumption about the distribution of indirect effect, confirms the Sobel test as well. The indirect effect for telework resources on job performance via on-the-job embeddedness was 0.09 (95% CI [0.04, 0.14], 95% CI [0.03, 0.14]; Table 3; Figs. 6, 7). Because these CIs did not contain zero, hypothesis 7 and 8 was thus supported.

**Table 3:** Mediation model test statistics.

Dependent variable	Sobel’s test t	Bootstrap (95% CI)	
Job performance		LLCI	ULCI
Mediator			
On-the-job embeddedness	3.57***	.04	.14
Off-the-job embeddedness	3.50***	.03	.14

*p < .05; **p < .01; ***p < .001.

## Discussion

### Theoretical implications

This study makes specific theoretical innovations, including two: First, instead of using turnover as the dependent variable, we present the results of job embeddedness in terms of job performance. There are three reasons here. The first reason is that in the context of COVID-19, most employees do not want to leave their jobs voluntarily, so job performance may become the only outcome of their efforts after excluding this option. The second reason is that we have used a cross-level model; from the leaders' perspective, job performance is also a goal they are focused on. The third reason is that the presentation of employee performance is the core exogenous variable that reflects employee-level behavior, whether voluntarily separated or terminated. This relatively objective indicator will provide more accurate feedback on the outcome of telework than their internal willingness to leave.

Second, in the context of COVID-19, the work scenario has shifted. The change of venue from company to home exacerbates the possibility of off-the-job embeddedness, which formal studies have yet to focus on. Drawing on family-work conflict theory, we enhance the explanatory power of off-the-job embeddedness by arguing that it may exacerbate family-work conflict, thereby reducing employee job performance to some extent (hypothesis only). However, off-the-job embeddedness does not change the likelihood of voluntary separation. That is, this paper explores the results of the possible impact of off-the-job embeddedness on job performance without changing the core premise of job embeddedness theory (that increasing on-and-off job embeddedness decreases the rate of voluntary turnover). This may contribute to the desire for firms to understand employees' off-the-job embeddedness due to work-family conflict and the inevitability of such activities. A decline in job performance is not a provocation by the employee to the company or the leader. Companies should provide some understanding or assistance. Secondly, it is significant for employees who are tired of family conflicts to know how to balance the on-and-off job embeddedness and keep their jobs with good performance.

Through our study, we further enrich this theoretical meaning:

1. By examining the moderating role of digital leadership, this paper illustrates the key means of ensuring job performance in remote work. In contrast, in traditional leadership studies, the focus has been more on leadership itself or responsible behavior. Although these studies validate the positive moderating effect of leadership power on job autonomy, they consider the limited penetration of leadership in remote work and the inconsistency between leadership activities and employee activities in time and space. These cannot answer the mechanisms of the future role of leadership after the normalization of the new crown epidemic.
2. This study extends the links between JD-R (Job Demands-Resources) theory and job embeddedness theory by revealing the path of telework resources-demands → on-and-off the job embeddedness → job performance across levels. Interestingly, we find that the linkage of off-the-job embeddedness → job performance is positive. This is contrary to our hypothesis but still consistent with some of the research on job embeddedness theory. Many scholars believe that both on-and-off the job embeddedness reduce employee turnover. Moreover, this paper translates the objective manifestation of reduced turnover rate into job performance. Therefore, the increase in job performance caused by off-the-job embeddedness remains scientific. Of course, most of the existing job embeddedness studies have focused on the relationship between job embeddedness and its outcome, and the construction of antecedent models for job embeddedness still needs to be improved. In this paper, we answer the possibility of such an antecedent model by introducing the JD-R theory. It is essential to focus on the fact that the particular telework context brought about by COVID-19 causes individual employee differences in practice. These differences will directly or indirectly affect the formation and development of job embeddedness, and the research results will give managers more excellent practical and policy guidance. Job embeddedness continues to affect job performance. Although the increased work demands amplify employees' embeddedness in their families and communities, they may be hoping for just such a situation: a free telecommuting atmosphere that makes them not want to return to the offline office. They may be frantic about accepting specific telecommuting requirements that allow companies to agree to allow them to stay in their homes or communities. However, they are not giving up their jobs because they do not want to lose them in an age full of uncertainty.
3. The robustness effect of job insecurity. Indeed, the moderating effect of job insecurity complements our hypothetical revision of the off-the-job embeddedness → job performance link. The preciousness of work is reinforced in a complex and volatile epidemic environment. Job insecurity becomes a stabilizer of job performance. At some level, job insecurity amplifies the positive relationship between off-the-job embeddedness and job performance despite employees being embedded outside of their jobs. Thus, the moderating effect of job insecurity is more pronounced in the conventional path of job embeddedness → job performance.

**Figure 8:**
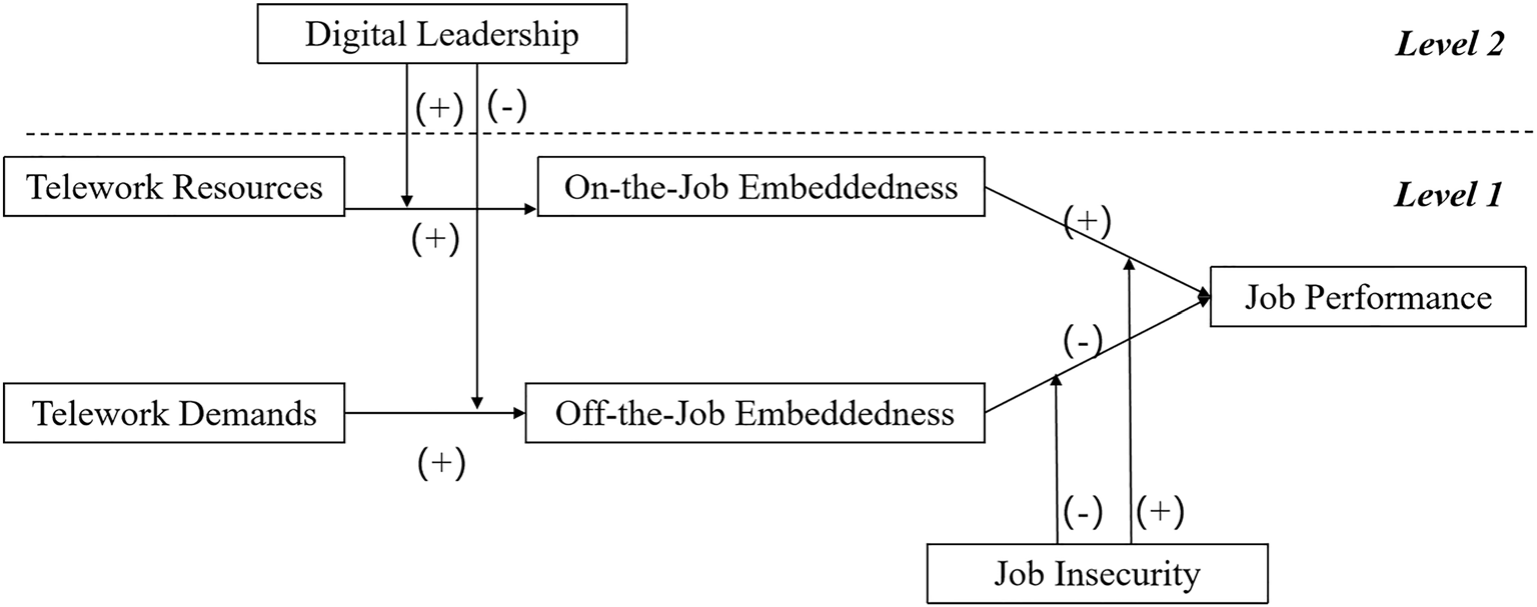
Actual theoretical path after verification.

Our study dispels corporate concerns about telework and reveals that in some specific cases, telework helps employees produce superior performance. We finally formed the empirical path as shown in Fig. 8.

### Management insights

Our findings suggest that telework does not decrease job performance during COVID-19. Traditional research on work-embeddedness-job performance relationships needs to catch up to theoretical work on this reality of the relationship. The subtle perspective of digital leadership and job insecurity in the context of telework can better substantiate the mechanism by which telework performance outcomes do not decline.

1. Telecommuting resources amplify job embeddedness, and telecommuting requirements expand job embeddedness. This is consistent with the Job Requirements-Resources (JD-R) theory. This suggests that the characteristics of telecommuting jobs are consistent with the traditional nature of work and do not result in a fundamental change in the resource endowment of work due to changes in the workplace. However, in conjunction with family conflict theory, telecommuting does have the potential to amplify family conflict. Either this relationship may have little impact in a traditional work setting. For some employees who value family and are more embedded outside of work, this may be why they prefer telecommuting: they can now move about their homes openly. They can convert some telecommuting requirements into reasons for their dependence on family and society. This is consistent with how some Chinese school students behave: they stay at home with the help of online class requirements and enjoy the comfort of learning that comes with home. Of course, this also causes conflicts at home, such as parents' complaints about their reduced learning effectiveness. The excessive embedding of employees outside of work likewise affects family disharmony: elders or spouses, for example, express dissatisfaction with their lethargic behavior, such as staying home for long periods and using work excuses to eliminate family chores. The realistic implication for business managers is that they should regulate the balance of telecommuting requirements and resources. They can use telecommuting resources to help employees better integrate into their work. Even if employees have to work from home because of COVID-19, they can still grow. Managers should regulate telecommuting requirements, and for those employees who use the home office as an excuse, managers can directly specify work requirements. Avoid using work as an excuse to deliberately cause conflicts at home and form a lethargic work atmosphere. For certain employees who value work but have to be restricted by family disputes, managers should increase the flexibility of work requirements, thus helping them to take time off from their families. This indirectly guarantees the comfort of work.
2. Related to this, we have verified the influence of digital leadership on the adjustability of embedding in and out of work. For employees embedded in their work, a higher role of digital leadership can help eliminate the differential impact of telework resources. Lower telework resources often perceive less digital leadership. For employees embedded outside work, digital leadership has a negative regulatory effect. Low digital leadership tends to lead to high out-of-work embedding. Higher telework demands will expand the perceived difference of digital leadership. We encourage managers to explore such interventions. We found that digital leadership has long-term positive benefits for telecommuting activities. Digital leadership has expanded the scope of attention of senior management to employees, gradually expanded the boundaries of employees' interests and atmosphere, and promoted the organization's sustainable development. It is recommended that enterprises actively train leaders' digital capabilities, open up more digital platforms, and meet the needs of employees. Encourage leaders to use digital activities to guide employees to understand and accept the values of the enterprise, give more care to employees in the epidemic, and enlarge the resource capacity of remote office.
3. Actively use the intermediary mechanism of job embedding. Increase the work embeddedness level of employees during the COVID-19 epidemic. Utilize various benefits, special allowances, and flexible telecommuting mechanisms. Use remote office resources to expand in-work embedding. Formulate work-family balance policies to reduce potential role conflicts among employees. Build a supportive cultural atmosphere, encourage employees to actively obtain support from communities and families under the requirements of a remote office, and optimize the positive aspects embedded outside the work. Expand the digital contact between managers and employees with the two-way adjustment mechanism of digital leadership and job insecurity. Managers give employees more trust, work autonomy, and family support resources and optimize their role behavior. Although out-of-work embedding may trigger the family crisis, it alleviates the potential impact of telework requirements on work performance: the warmth and resources of the family may neutralize the tedious requirements of telework. The connection between family and community may also expand the need for employees to improve their work performance. The sense of job insecurity further magnifies the anxiety of employees about “epidemic survival” and ultimately leads to an increase in work performance.

It should be noted that in the case of normalization of the epidemic, it is likely that a large number of employees will face the possibility of multiple infections. Intermittent telecommuting will not cause an enterprise crisis. Enterprises can also refer to the contribution of this article to develop opportunities for telecommuting for future employees with higher demands for freedom and office flexibility. For example, some IT professionals and designers may perform more work in a remote environment. We hope this paper can change China's or developing countries' rigid working mechanisms. The epidemic will eventually end, but the future of telecommuting is measurable. We hope that the resources and requirements of telecommuting will lead to the adjustment of the work embedding mechanism and enlarge the work performance of the enterprise. At the same time, excessive workplace investment will inhibit the development of many small and micro enterprises. Small and micro enterprises may abandon the traditional work scene and produce more initial results with the workroom or family-style work mechanism.

## Methods

### Sample and procedures

In early March 2022, an outbreak of COVID-19 in Shanghai led to nearly 3 months of closed-off management on the social level. As a result, the majority of employees working in Shanghai experienced a form of telecommuting. In the future, most corporate employees will still face the challenge of telecommuting. In this paper, we take the EMBA students of Tongji University School of Economics and Management as the research subjects. Most of these participants hold leadership positions in their companies (e.g., supervisors, managers, etc.). They have their subordinates and teams. Most of the companies in which the participants worked were located in Shanghai and involved different industries, which provided a rich and heterogeneous sample for our study. The authors of this study obtained this data by serving as teaching assistants in these courses.

The purpose, procedures, and confidentiality of this study were clearly described to the participants prior to conducting the survey, and we received generous support and cooperation from the participants. We invited 40 team supervisors to evaluate their members’ job performance and send research questionnaires to their members. Of 352 team members, 328 from 36 teams responded. After the data collection, we filtered the data and deleted the problematic ones (e.g., incompletely-answered questionnaires). The valid sample consisted of 308 team members from 32 teams, resulting in final response rates of 87.5% for team members and 88.89% for team supervisors. Among the 308 team members, 51% were female; 27.50% had a bachelor's degree and above; the average age was 34.81 years (*SD* = 7.68); Among the 32 teams, the average team size was 9.63 persons (*SD* = 3.12).

### Measures

We will take advantage of the time between and after classes to conduct informal and formal interviews with the participants. The interviews aimed to understand the participants' perceptions and understanding of the resources-demands of teleworking and digital leadership. In structured interviews, we designed an interview outline based on classic work demands-resources theory, leadership theory, and work embeddedness theory as a basis for understanding whether these variables present new characteristics in telecommuting contexts. The ultimate goal is to form initial scales about telecommuting resources-demands, on-and-off job embeddedness, job performance, job insecurity, and digital leadership and to form research questionnaires through EFA and CFA methods. We will show the full scale in the "Appendix".

We obtained approval and permission for the questionnaire data from the Committee of the School of Economics and Management of Tongji University (2023-0016). All questionnaire experiments were conducted according to the rules and regulations. This study does not involve human tissue samples or other human experiments. During the questionnaire release process, informed consent was observed from all participants. In addition, this study did not involve participants under the age of 18.

#### Telework demands-resources

We measured telework demands-resources using a modified version of the measure created by Gonzalez‐Mulé^72^, Demerouti e al.^73^. Sample telework demands items included, “To what extent do you agree that telework requires working very hard?”, “To what extent do you agree that telework requires long periods of intense concentration?”. Sample telework resources items included, “I can make my own decisions about many things at telework.”, “Telework gives me the opportunity to learn new things.”. Following Swider and Porter^74^, we standardized and aggregated the items to create measures of telework demands (9 items; α = 0.96) and off-the-job embeddedness (9 items; α = 0.95).

#### Digital leadership

We measured digital leadership using a modified version of the measure created by C. Meier et al.^75^ and Banks et al.^31^. Sample items included, “The leaders in our company recognizes the network character by identifying the competencies and contacts of individual employees.” and “Leaders have high confidence in the capabilities because of the fast changing environment”. We standardized and aggregated the items to create measures of on-the-job embeddedness (6 items; α = 0.93).

#### Job embeddedness

We measured on-and-off the job embeddedness using a modified version of the measure created by Burrows et al.^65^. Sample on-the-job embeddedness items included, “I am in close contact with my colleagues at work or in life,” “I get along well with my colleagues in my work or life,” and “I have similar values to most of my colleagues”. Items were modified to include the context of COVID-19 and telework environment. Sample off-the-job embeddedness items included, “My spouse works outside the home,” “I really love the place where I live,” and “If I were to leave this community, I would miss my nonwork friends”. We standardized and aggregated the items to create measures of on-the-job embeddedness (14 items; α = 0.96) and off-the-job embeddedness (11 items; α = 0.95).

#### Job insecurity

We measured job insecurity using a modified version of the measure created by Mauno et al.^76^ Sample job insecurity items included, “My job is insecure,” and “I am worried about the possibility of being fired”. We standardized and aggregated the items to create measures of job insecurity (5 items; α = 0.94).

#### Job performance

We measured job performance using a modified version of the measure created by Jansen et al.^77^ Sample job insecurity items included, “The employee demonstrates expertise in all job-related tasks.,” and “The employee fulfills all the requirements of the job”. We standardized and aggregated the items to create measures of job performance (5 items; α = 0.91).

### Informed consent 

Informed consent was obtained from all subjects involved in the study.

### Institutional review board statement

All participants were informed and consented to participate.

### Supplementary Information

Supplementary Information.

## Data Availability

The data presented in this study are available on request from the corresponding author.
