# Supplementary material for: The impact mechanism of telework on job performance: a cross-level moderation model of digital leadership
Source: Sci Rep. 2024 May 31;14:12520. doi: 10.1038/s41598-024-63518-6 (PMC11143337; doi:10.1038/s41598-024-63518-6)
Supplement: Supplementary file 1 — Supplementary Information. [file 41598_2024_63518_MOESM1_ESM.docx]

**APPENDIX**

| ***Telework Demands***  *Modified Job Demands Scale Based on Gonzalez‐Mulé et al.(2017)* |
| --- |
| To what extent do you agree that your job requires working very hard? |
| To what extent do you agree that your job requires working very fast? |
| To what extent do you agree that your job requires long periods of intense concentration? |
| To what extent do you agree that your job is very hectic? |
| To what extent do you agree that you have too much work to do everything well on your job? |
| To what extent do you agree that you are not asked to do an excessive amount of work at your job? (reverse scored) |
| To what extent do you agree that you have enough time to get the job done? (reverse scored) |
| To what extent do you agree that do you agree that you are free of conflicting demands that others make on your job? (reverse scored) |
| How frequently does your job require working under time pressure? |
| ***Telework Resources***  *Modified Job Resources Scale Based on Demerouti E et al.(2001), Karasek Jr R A(1979)* |
| I can make my own decisions about many things at work. |
| I have a say in what happens at work. |
| I have the right to decide how to do my job. |
| My job gives me the opportunity to learn new things. |
| I often accept tasks that promote my development and strengthen my skills . |
| My job gives me the possibility to develop and improve fully. |
| I can rely on the help of my superiors and colleagues when I encounter difficulties in my work. |
| I often get cooperation and support from the people I work with, which helps me a lot in my work. |
| The status of my relationship with my colleagues and superiors has a great impact on my work. |
| ***Digital leadership***  *Modified Digital Leadership Scale Based on C. Meier, et al.(2017), George C. Banks et al.(2022)* |
| The leaders in our company recognizes the network character by identifying the competencies and contacts of individual employees. |
| In our firm, leaders develop trust in the employees. |
| In our firm, leaders provide necessary information to employees. |
| In our firm, leaders act as learning guides and coaches. |
| Leaders are open to critique, feedback and new ideas. |
| Leaders have high confidence in the capabilities because of the fast changing environment. |
| ***On-the-job embeddedness***  *Modified On-the-job Embeddeness Scale Based on Mitchell, T. R. et al. (2001), Porter, C. M. et al.(2019)* |
| I am in close contact with my colleagues at work or in life. |
| I get along well with my colleagues in my work or life. |
| I have similar values to most of my colleagues. |
| My current job makes full use of my skills and talents.. |
| I am satisfied with the organization of my work in terms of time, content, and manner. |
| My personal values are well reflected in this company. |
| I think the authority given to me by the unit is equal to the responsibility I have assumed. |
| I fit well into the organizational culture of this company. |
| I can receive compensation commensurate with my performance. |
| I have a clear career path in this organization with great opportunities for advancement. |
| I am satisfied with the benefits and allowances provided by this organization. |
| The development prospect of this organization is very good, and it is beneficial for me to choose to continue working here. |
| I have a lot to lose if I don't do this job. |
| ***Off-the-job embeddedness***  *Modified Off-the-job Embeddeness Scale Based on Lee et al. (2004)* |
| I’m not married.a (reverse coded) |
| My spouse works outside the home. |
| Do you own the home you live in? |
| I really love the place where I live. |
| The weather where I live is suitable for me. |
| This community is a good match for me. |
| I think of the community where I live as home. |
| The area where I live offers the leisure activities that I like. |
| Leaving this community would be very hard. |
| If I were to leave this community, I would miss my nonwork friends. |
| Having to give up my home to relocate would be very difficult. |
| If I were to leave the community, I would miss my daily routine. |
| If I were to leave the community, I would miss my neighborhood. |
| ***Job Insecurity***  *Modified Job Insecurity Scale Based on Mauno,S. et al.(2001)* |
| My job is insecure. |
| My job is likely to change in the future. |
| My job is not permanent. |
| I am worried about the possibility of being fired. |
| The thought of getting fired really scares I. |
| ***Job Performance***  *Modified Job Performance Scale Based on Jansen, A. et al.(2013)* |
| The employee demonstrates expertise in all job-related tasks. |
| The employee manages more responsibility than typically assigned. |
| The employee fulfills all the requirements of the job. |
| The employee achieves the objectives of the job. |
| The employee plans and organizes to achieve objectives of the job. |

***Note.*** Responses provided using seven-point Likert scale from strongly disagree—strongly agree.
